# Why Varicoceles Recur: Missed Venous Anatomy and Contemporary Strategies for Salvage

**DOI:** 10.3390/jcm15041524

**Published:** 2026-02-14

**Authors:** Aris Kaltsas, Nikolaos Sofikitis, Fotios Dimitriadis, Athanasios Zachariou, Michael Chrisofos

**Affiliations:** 1 Third Department of Urology, Attikon University Hospital, School of Medicine, National and Kapodistrian University of Athens, 12462 Athens, Greece; ares-kaltsas@hotmail.com; 2 Department of Urology, Faculty of Medicine, School of Health Sciences, University of Ioannina, 45110 Ioannina, Greece; nsofikit@uoi.gr (N.S.); zahariou@otenet.gr (A.Z.); 3 First Department of Urology, Faculty of Medicine, School of Health Sciences, Aristotle University of Thessaloniki, 54124 Thessaloniki, Greece; difotios@auth.gr

**Keywords:** varicocele, recurrence, persistence, redo varicocelectomy, microsurgery, embolization, sclerotherapy, male infertility, Doppler ultrasonography, venography

## Abstract

**Background/Objectives:** Varicocele repair can improve semen parameters and pregnancy rates in appropriately selected men; however, persistence or recurrence remains a common cause of treatment failure with ongoing infertility or scrotal pain. Because mechanisms and definitions vary across studies, counseling and salvage selection can be challenging. This review synthesizes contemporary evidence on why varicocele recur and provides an anatomy-informed approach to evaluation and retreatment. **Methods:** A narrative evidence synthesis was performed using PubMed/MEDLINE, prioritizing clinical practice guidelines, systematic reviews, meta-analyses, and contemporary adult and adolescent clinical series addressing mechanisms of failure, diagnostic workup, and outcomes of salvage microsurgery and endovascular therapy. **Results:** Recurrence rates vary by technique and follow-up, with the lowest rates reported in contemporary microsurgical subinguinal series. The dominant drivers of failure are incomplete venous control and complex reflux pathways, including duplicated internal spermatic veins and missed collaterals such as cremasteric, external spermatic, gubernacular, and deferential veins. Clinical examination remains central; Doppler ultrasonography is most useful when pain persists or semen parameters and testicular growth do not improve. Venography can define culprit channels in complex or multiply treated cases and enables targeted embolization. Retreatment achieves high anatomic success with consistent improvements in semen parameters and meaningful pregnancy rates in available series, with modality-specific complication profiles. **Conclusions:** Recurrent varicocele should be managed with structured reassessment that links venous anatomy and the index procedure to the salvage option. Microsurgical redo is generally favored after non-microscopic repairs, whereas endovascular occlusion is often preferred after prior surgery or when venographic mapping is needed.

## 1. Introduction

Varicocele is defined as pathological dilation of the veins of the pampiniform plexus and is frequently encountered in both adolescents and adults. Epidemiological estimates suggest that it affects at least 15% of adolescent males and more than 30% of men evaluated for infertility [1,2]. In appropriately selected men with palpable varicocele and abnormal semen parameters, repair improves sperm quality and increases the probability of pregnancy, although the magnitude of benefit varies between couples [3,4,5]. Spontaneous conception is not assured, and a proportion of couples will still proceed to assisted reproductive technologies after intervention [6,7,8]. Current treatment options include open or laparoscopic surgery and percutaneous endovascular techniques [9].

Despite these benefits, persistence and recurrence remain clinically important causes of treatment failure and ongoing infertility or scrotal pain [10]. The term persistence is used for continued clinical or sonographic reflux after the index procedure, whereas recurrence is used for varicocele reappearance following an interval of apparent resolution. Notably, many publications do not distinguish these entities and use the terms interchangeably [11]. The primary aim of this narrative review is to clarify why varicoceles fail to resolve or recur by integrating missed venous anatomy with hemodynamic reflux patterns. The intent is to support clinical evaluation and the selection of salvage strategies rather than to issue a formal guideline. Reported recurrence rates remain heterogeneous because studies differ in patient age, indications, clinical grade, follow-up duration, diagnostic thresholds, and procedural technique [11].

Recurrence rates have been reported from near 0% up to 30% across studies [12]. Contemporary microsurgical subinguinal techniques report the lowest recurrence rates in large series [13,14,15], whereas non-microscopic high ligation or inguinal approaches have historically shown higher relapse [16,17,18,19]. Endovascular embolization demonstrates intermediate recurrence rates that depend on venous anatomy, embolic agent, and technical success [20,21,22]. A large U.S. claims analysis (2007–2014) found that 1.7% of men underwent a second varicocele repair within approximately two years of the initial procedure, although re-intervention rates likely undercapture clinically silent residual reflux [23]. In adolescents, both the index technique and the preoperative hemodynamic pattern of reflux may influence later failure, and delayed recurrences have been described on long-term follow-up [24].

Overall, most repairs are durable, yet recurrent or persistent varicocele remains clinically relevant given the volume of procedures performed and its potential impact on fertility and symptoms. Accurate detection depends on a meticulous standing examination, and interexaminer variability can influence reported outcomes [25]. Doppler ultrasonography can improve diagnostic confidence when symptoms persist or clinical findings are equivocal; however, the clinical meaning of low-grade, imaging-only residual reflux remains uncertain and may lead to overdiagnosis if applied indiscriminately [26]. This uncertainty should be acknowledged explicitly when counseling patients after repair and when deciding whether retreatment is warranted.

## 2. Methods

A narrative review was conducted focusing on mechanisms of persistence or recurrence after varicocele repair and on contemporary salvage strategies. PubMed/MEDLINE was searched from database inception to 1 February 2026, restricted to English-language human studies. Priority was given to clinical practice guidelines, systematic reviews, meta-analyses, randomized trials, and contemporary observational series in adults and adolescents reporting mechanisms or predictors of persistence or recurrence, post repair diagnostic pathways including clinical examination, Doppler ultrasonography, venography, and cross-sectional imaging when relevant, or outcomes of salvage microsurgery or endovascular treatment. Outcomes of interest included technical or anatomic success, complications, semen parameters, pain, and pregnancy. Case reports were generally excluded but were considered when illustrating uncommon contributors to recurrence such as renal vein entrapment. Search terms were used alone and in combination and included “varicocele”, “recurrence”, “persistence”, “redo”, “salvage”, “microsurgery”, “embolization”, “sclerotherapy”, “Doppler”, “venography”, and “nutcracker”. Reference lists of key reviews were screened to identify additional relevant studies. Evidence was synthesized qualitatively with emphasis on linking the likely failure mechanism to the most appropriate salvage option.

## 3. Mechanisms and Predictors of Varicocele Recurrence

Recurrent varicocele arises when retrograde venous flow persists after treatment because one or more channels of spermatic cord drainage remain patent. The most frequent substrate is an untreated branch of the internal spermatic vein that was not identified during the index repair. Even a slender tributary can progressively enlarge under sustained pressure and reconstitute reflux. High retroperitoneal branches are a typical source when low approaches fail to control the entire pathway [10,27].

**Figure 1:**
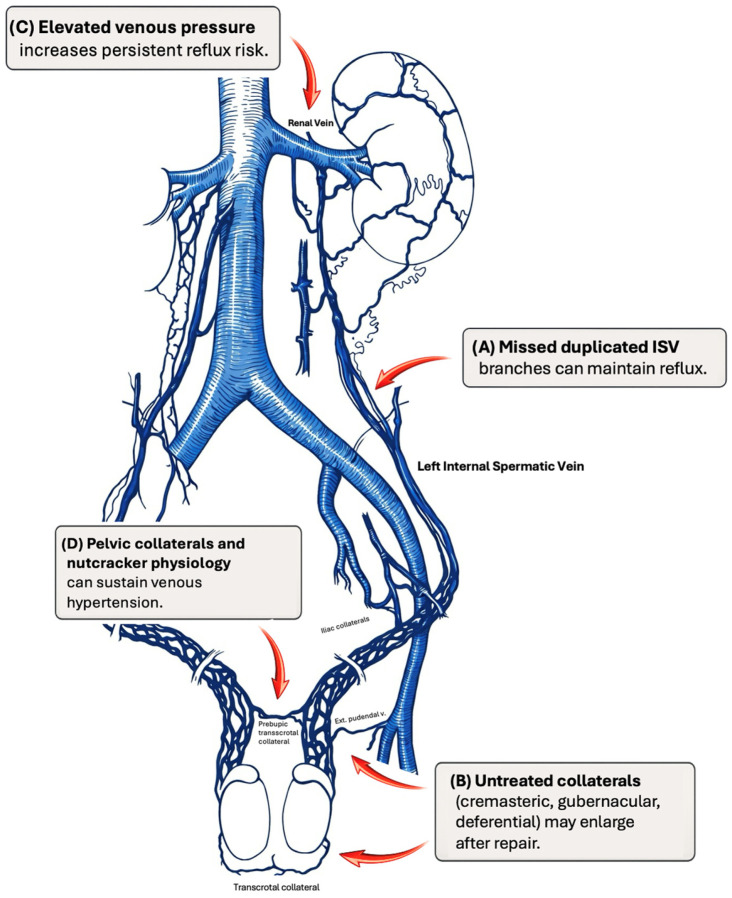
Simplified venous anatomy and common reflux pathways underlying persistence or recurrence after varicocele repair. Red arrows highlight the main reflux pathways/collateral routes. (**A**) Missed duplicated internal spermatic vein (ISV) branches can maintain reflux. (**B**) Untreated collateral drainage (cremasteric/external spermatic, gubernacular, and deferential veins) may enlarge after repair and sustain reflux. (**C**) Elevated venous pressure increases the risk of persistent reflux. (**D**) Pelvic collaterals and nutcracker-related physiology can sustain venous hypertension and contribute to persistence/recurrence.

Collateral routes may also maintain the varicocele circuit. External spermatic or cremasteric veins, gubernacular veins, and deferential veins can carry significant flow if they are not addressed at the time of repair. This is particularly relevant after high ligation techniques that focus on the internal spermatic vein alone. Some surgeons advocate testicular delivery during subinguinal repair to ligate gubernacular channels, although recent work indicates that routine treatment of these veins is not always necessary to prevent relapse [10,27]. Key venous pathways and commonly missed collateral routes that underpin persistence or recurrence are summarized in Figure 1.

Anatomical variation is a major determinant of failure. Duplication and complex branching of the internal spermatic vein are frequent findings, and unrecognized variants increase the likelihood of residual reflux. Radiographic classifications such as that of Bähren describe patterns with multiple renal vein ostia and collateral communications that predispose to incomplete cure when unappreciated [28]. Venovenous anastomoses may reroute blood into pelvic or lumbar systems, allowing a ligated main trunk to be bypassed through alternative channels. Postoperative venography in men with recurrence frequently demonstrates reconstitution of the pampiniform plexus via such pathways [27]. Prevention, therefore, depends less on the nominal ligation level and more on comprehensive identification and control of all refluxing channels while preserving the artery and lymphatics [28,29].

Beyond anatomic variants, the hemodynamic pattern of reflux may predispose to persistence or apparent recurrence when it is not identified preoperatively. The venographic classification introduced by Coolsaet distinguishes renospermatic reflux (type I), ileospermatic reflux (type II), and combined reflux (type III) [30]. From a practical perspective, type II and type III patterns imply clinically relevant reflux through deferential and or cremasteric systems in addition to, or instead of, the internal spermatic vein. In such cases, procedures that target the internal spermatic vein alone, including high ligation, may fail to eliminate all refluxing pathways [31].

In pediatric and adolescent cohorts treated laparoscopically, comprehensive preoperative Doppler mapping of refluxing systems and selective interruption of a refluxing deferential vein have been associated with lower rates of persistence and recurrence [32,33]. In a prospective adolescent study, shunt type varicocele was associated with a higher risk of developing hypotrophy during observation and with higher recurrence rates after retroperitoneal ligation, compared with an inguinal approach that included ligation of both internal and external spermatic veins [34].

Renal venous hypertension also shapes outcomes. Compression of the left renal vein (nutcracker physiology) increases the upstream pressure gradient and can favor persistence or recurrence even after technically sound venous interruption. This mechanism plausibly contributes to the higher recurrence observed in very lean men and should be considered when counseling patients with large left-sided varicoceles or disproportionate venous dilatation [12].

Age at treatment adds further nuance. In adolescents, technique selection has historically shaped both recurrence and complication profiles. A recent proportion meta-analysis in pediatric and adolescent cohorts reported recurrence between about two and eight percent across techniques, with higher hydrocele rates when lymphatics were not preserved during laparoscopy and lower hydrocele rates after embolo-sclerotherapy at the cost of more frequent technical access failure. A randomized trial in teenagers found that scrotal antegrade sclerotherapy was not inferior to laparoscopic Palomo for clinical recurrence and was associated with less postoperative discomfort. Earlier work noted that artery-sacrificing Palomo can minimize recurrence but increases hydrocele formation, an effect that modern lymphatic-sparing methods and microscopy can mitigate [35,36].

Associations between preoperative clinical grade and recurrence are inconsistent. Some contemporary series link higher grade to greater risk, while others find limited independent effect when modern microsurgery is applied. Divergent findings likely reflect differences in technique, follow-up intensity and the rigor with which collateral pathways were addressed [37,38].

Quantitative and temporal factors contribute as well. Prolonged Doppler reflux (≥4.5 s) and body mass index < 25 kg/m^2^ have been associated with higher recurrence [39,40]. Detection depends on timing: small untreated veins may take years to become clinically evident. In one adolescent cohort, new palpable recurrences appeared as late as 76 months despite a normal examination at a mean of 27 months after surgery [41]. Early ultrasound studies suggest that persistent reflux on the first postoperative day may predict later recurrence, yet the clinical value of identifying subclinical reflux remains uncertain [26]. Standardized criteria for defining and monitoring recurrence are needed to clarify which residual findings are clinically meaningful for fertility and which can be safely observed.

Recurrence rates are often presented as technique-specific, yet interstudy comparisons are highly sensitive to heterogeneity in surgeon experience, use of magnification (loupes versus microscope), learning curve effects, the definition of recurrence (clinical versus Doppler-defined), and the rigor and duration of follow-up. Reported outcomes may also be influenced by whether the index evaluation captured the hemodynamic type of varicocele and, consequently, whether deferential or cremasteric reflux pathways were addressed at the primary procedure. With these caveats, contemporary syntheses generally show the lowest recurrence after microsurgical subinguinal repair, higher recurrence after non-microscopic high ligation, and intermediate rates after laparoscopy and embolization. These gradients likely reflect differences in the extent to which each approach enables systematic control of internal spermatic and accessory reflux pathways and is applied to an appropriately characterized varicocele [11,38,42]. Table 1 summarizes typical rates across modalities.

The principal modifiable predictor of failure is the completeness of venous control at the index operation. Series with higher recurrence commonly involved repairs performed without magnification or with limited attention to accessory outflow pathways. Residual reflux through cremasteric, external spermatic or small internal spermatic branches accounts for many apparent recurrences, a pattern consistently identified in systematic evaluations of failed cases [11].

In practice, these risk factors inform both initial planning and salvage strategy. A lean man with a large left varicocele warrants assessment for nutcracker physiology and counseling about a higher risk of relapse even after high-quality surgery. Complex venous patterns on venography favor a complementary plan that combines microscope-guided ligation of accessible channels with targeted endovascular occlusion of high retroperitoneal collaterals. Across scenarios, comprehensive treatment of all refluxing pathways with preservation of the artery and lymphatics remains the unifying principle for durable results [12,51].

## 4. Impact of Recurrent Varicocele on Fertility and Testicular Function

Recurrent varicocele perpetuates the biological stresses that characterize the primary lesion. Ongoing venous reflux maintains scrotal hyperthermia, tissue hypoxia, and heightened oxidative stress, and it may expose the testis to refluxed renal and adrenal metabolites. The combined effect disrupts the seminiferous epithelium, damages sperm membranes and DNA, and impairs endocrine function, thereby undermining any gains achieved after the index repair. Contemporary reviews consistently identify oxidative stress, heat stress, and hypoxia as central mechanisms of testicular injury in varicocele, with accumulating molecular evidence linking these pathways to defective spermatogenesis and sperm DNA damage [52,53].

Failure to abolish reflux is a recognized cause of post-varicocelectomy treatment failure. In men who re-present with a palpable or symptomatic recurrence, semen quality often remains suboptimal, and fertility outcomes are compromised until the lesion is definitively corrected. A systematic review of 1073 men with persistent or recurrent varicocele found that repeat treatment achieved anatomic success in most series and was associated with meaningful clinical improvements: semen parameters improved in the great majority of studies, pain resolved in more than ninety percent of cases, and pregnancy occurred in roughly one-sixth to more than half of couples within twelve months, despite heterogeneity in populations and techniques. These data reinforce that active management of recurrence can restore the benefits seen after primary repair [54].

Across adult cohorts, recurrent disease is typically accompanied by depressed sperm concentration, motility, and morphology compared with men whose first repair remained durable. Salvage treatment tends to reverse a significant portion of this deficit. In the systematic review above, most series documented post-intervention gains in standard semen measures after redo repair, aligning with broader meta-analyses in primary disease that show consistent enhancement of sperm count and motility following correction of reflux. The biological plausibility of these gains is supported by meta-analytic evidence that varicocele repair reduces sperm DNA fragmentation, a marker tightly linked to oxidative stress and reproductive success. Recent syntheses indicate that men with clinical varicocele have significantly higher DNA fragmentation than controls and that repair lowers DNA fragmentation, with reductions sustained on follow-up [54,55].

Endocrine recovery is also relevant. Varicocele can impair Leydig cell function in a subset of men. A recent meta-analysis that pooled nearly fifty studies reported a significant rise in total testosterone after varicocele repair compared with preoperative levels and compared with men who did not undergo repair, with accompanying favorable shifts in gonadotropins. Although dedicated analyses in redo populations remain limited, the same physiology applies to recurrent lesions, and durable abolition of reflux would be expected to stabilize or improve androgen production in responsive individuals [56].

Sperm DNA integrity sits at the interface of basic physiology and clinical outcomes. Meta-analytic data demonstrate that varicocele is associated with higher DNA fragmentation indices and that repair reduces these indices, supporting a mechanistic link between relief of oxidative stress and improved gamete quality. This molecular signal is clinically relevant because elevated DNA fragmentation is associated with lower natural conception rates and with suboptimal assisted reproductive outcomes. Taken together, correction of a recurrent varicocele is likely to lower DNA fragmentation in the same direction as primary repair, thereby improving the probability of conception either naturally or with assisted methods [55].

Fertility endpoints are central for couples considering salvage. In the systematic review of persistent and recurrent disease, post-treatment pregnancy rates ranged from seventeen to fifty-eight percent at one year, reflecting variation in female factors and baseline semen quality but nonetheless indicating that a substantial proportion of couples conceive after effective retreatment. Beyond spontaneous conception, several recent syntheses suggest that repairing an active varicocele can improve outcomes of assisted reproduction. Meta-analyses focused on men proceeding to intracytoplasmic sperm injection report higher clinical pregnancy and live birth after prior varicocele repair than after proceeding directly to ICSI, although study quality varies and further trials are needed. These findings support counseling that definitive management of recurrence can enhance the prospects of both natural and assisted conception [54,57,58].

Adolescent considerations differ in emphasis but point in the same direction. Untreated reflux in youth can impair ipsilateral testicular growth, while timely correction allows catch-up growth in a large proportion of boys with preoperative asymmetry. A comprehensive pediatric review summarizing a meta-analysis of fourteen studies reported catch-up growth rates commonly between sixty and ninety percent after varicocelectomy, and additional series show catch-up after percutaneous embolization in young adults. At the same time, contemporary cohort data indicate that many adolescents with hypotrophy also demonstrate catch-up during observation, underscoring the need to individualize indications for intervention and to monitor growth trajectories closely [59,60,61].

Pain is another clinically important domain. In men treated for recurrent or persistent varicocele because of orchialgia, symptom control exceeds ninety percent in pooled analyses, indicating that definitive retreatment can deliver durable relief alongside potential reproductive benefits [54].

Overall, recurrent varicocele sustains the same injurious microenvironment as the primary lesion and thereby compromises spermatogenesis, sperm genomic integrity, and in some men endocrine function. Contemporary evidence, anchored by systematic reviews and meta-analyses, supports the view that eliminating persistent reflux through a well-executed salvage procedure restores a substantial portion of lost function, improves semen quality, reduces sperm DNA fragmentation, and can increase both natural pregnancy and the effectiveness of assisted reproduction in appropriately selected couples [54,55,56].

## 5. Diagnostic Challenges in Recurrent Varicocele

Recurrent varicocele is a recognized cause of treatment failure after varicocelectomy, with recurrence rates reported as low as five percent in contemporary microsurgical series and as high as thirty percent with older techniques [11]. Clinical presentation mirrors primary disease. Men often report a dull scrotal heaviness or ache that may progress to persistent pain. On examination, a recurrent lesion is a compressible serpiginous plexus that refills with a Valsalva maneuver. Postsurgical fibrosis within the spermatic cord can confound palpation, so the examiner must distinguish firm scar tissue from persistent dilated veins. Subtle or early recurrences require a meticulous standing examination performed in a warm room with repeated Valsalva efforts to relax the cremasteric reflex. In adolescents, recurrence is particularly relevant because of the risk of impaired testicular growth and future fertility. Follow-up should document testicular volume and symmetry, since semen analysis is often unavailable in this age group. Failure of the affected testis to demonstrate catch-up growth after repair should prompt evaluation for persistent reflux. Persistent scrotal pain after varicocelectomy also warrants reassessment because recurrent varicocele is a common and remediable cause of postoperative orchialgia [59,62,63].

Color Doppler ultrasonography is the principal adjunct for confirmation and characterization of suspected recurrence. Ultrasonography provides objective evidence of venous dilatation and retrograde flow during Valsalva when the physical examination is equivocal. Standardized recommendations from the European Society of Urogenital Radiology advise measurement in the upright position with Valsalva and recognition of venous reflux of more than two seconds. Most clinicians use a largest venous diameter of at least three millimeters as the diagnostic threshold. Comprehensive scanning above and below the original ligation level is essential to reveal collateral pathways that may have been missed at the index procedure. Failure to interrogate high retroperitoneal or pelvic collaterals can yield false negative studies, whereas brief low-volume reflux in small residual veins can produce false positive impressions that lack clinical significance [64].

Routine imaging after repair is not recommended in asymptomatic men, because detection of low-grade residual reflux can increase reintervention rates without clear evidence of benefit [2,65]. Ultrasonography is most useful when infertility or pain persists, when the physical examination is equivocal, or when testicular growth fails to improve in adolescents. In these contexts, targeted Doppler assessment can document reflux, identify potentially missed venous districts (including deferential or cremasteric systems), and help stratify whether a clinically meaningful recurrence is present [2,66]. Closer surveillance may be justified in adolescents with persistent or progressive testicular volume asymmetry on serial measurements, and in men whose semen parameters fail to improve within the expected postoperative timeframe, particularly when the preoperative hemodynamic pattern suggested mixed outflow pathways [67,68,69].

In practice, a recurrence is considered clinically meaningful when it is palpable or visible and correlates with symptoms, testicular dysfunction, or impaired semen parameters. Subclinical recurrences identified only by imaging remain of uncertain importance and are typically observed unless reproductive goals remain compromised [70]. In adolescents, close observation of testicular growth and function is central to decision-making. Intervention is generally reserved for significant testicular asymmetry, persistent pain, or abnormal semen analysis when available [71].

Anatomic clarification becomes more important after prior surgery or when ultrasound does not explain symptoms. Venography can delineate duplicated trunks, pelvic/inguinal duplications, and collateral communications and can facilitate immediate embolization. Nonetheless, selective internal spermatic venography alone may miss recurrence mechanisms when reflux is primarily extrafunicular. In one study of men with persistent varicocele and abnormal semen parameters after conventional surgery, scrotal Doppler demonstrated reflux in all cases, whereas selective internal spermatic venography confirmed reflux via the internal spermatic vein in only 22%, implying that many recurrences were maintained by non-internal-spermatic routes [72]. For this reason, venography is best positioned as a problem-solving tool rather than a routine test, reserved for equivocal cases, suspected proximal or pelvic collaterals, or recurrences after technically sound microsurgery in which further cord exploration would be high risk or low yield [51].

## 6. Management Strategies for Recurrent Varicocele

A clear statement of therapeutic goals is the foundation of retreatment. The priorities may be restoration of fertility, relief of pain, or both, and the plan should account for the severity of recurrence and the index technique. Contemporary evidence supports active management. A systematic review of more than one thousand men with persistent or recurrent disease reported high rates of anatomical success that ranged from roughly sixty to one hundred percent, with pregnancies recorded in about seventeen to fifty-eight percent within one year and improvements in semen parameters in most series. These findings indicate that retreatment is often worthwhile when a clinically significant recurrence is present [54].

### 6.1. Microsurgical Redo Varicocelectomy

Subinguinal microsurgical ligation remains the reference technique because it allows meticulous identification of internal spermatic vein trunks and small collaterals while preserving the artery and lymphatics. This approach is well suited to salvage after nonmicroscopic repairs in which small branches may have been missed. In the largest dedicated series, redo subinguinal microsurgery achieved complete clinical resolution after failure of high ligation and yielded meaningful gains in semen quality. Pregnancy was achieved by a large proportion of couples and the need for assisted reproduction declined when compared with observation [73,74].

More recent pooled evidence confirms these trends. A meta-analysis focused on redo surgery documented significant increases in sperm concentration, progressive motility, total motile count and normal morphology, with an overall pregnancy rate near one-third across included studies. Complication rates remained low and broadly comparable to those seen with primary microsurgery [75].

The operative field in recurrent cases often contains scar from the index operation. A subinguinal route allows dissection distal to prior high ligation and facilitates control of cremasteric and external spermatic veins when they contribute to reflux. When the index procedure was already a subinguinal microsurgical repair, surgeons may consider a slightly higher inguinal exposure or switch to an endovascular approach if dense fibrosis obscures the field [73].

### 6.2. Laparoscopic or High Retroperitoneal Ligation

Laparoscopy offers a vantage point above prior low repairs and permits division of gonadal veins at a proximal level near their origin. Success depends on careful identification of all venous channels and on disciplined lymphatic and arterial preservation. Contemporary summaries show variable recurrence after laparoscopy, from very low figures in expert series to rates in the low double digits when collateral pathways are not treated. This heterogeneity reflects differences in technique and experience rather than an inherent limitation of the approach [35].

Open high ligation can be used when resources or expertise limit other options, especially in adolescents at centers where this remains standard practice. The strategy may reduce relapse when meticulously performed, though the risk of postoperative hydrocele is higher if lymphatics are not spared [76].

### 6.3. Percutaneous Embolization and Sclerotherapy

Endovascular therapy is highly attractive in the salvage setting because it couples diagnostic mapping with targeted occlusion in a single session. Venography in men who fail surgery frequently reveals anatomic explanations for persistence, including duplication of the gonadal vein and unrecognized collaterals. In such cohorts, technical success of embolization approaches 100% and major complications are rare [77].

Across modern series and reviews, endovascular treatment achieves technical success rates consistently above ninety percent with favorable safety. Inability to catheterize the target vein remains the main cause of technical failure and is variably reported from about eight percent to as high as thirty percent in anatomically challenging cases, a range that underscores the value of experienced operators [78,79].

Choice of embolic agent can be tailored to anatomy. Coils and plugs provide mechanical occlusion, foamed sclerosants penetrate small channels, and cyanoacrylate glues offer rapid permanent sealing of complex plexuses. Evidence drawn from pooled analyses shows that clinical efficacy is high for each of these strategies when venography guides comprehensive occlusion of all contributing channels [78].

Antegrade scrotal sclerotherapy is a surgical–radiological hybrid performed through a short scrotal incision with fluoroscopic confirmation of distribution. Large contemporary series document low persistence and meaningful improvement in semen counts, and randomized and comparative studies in adolescents demonstrate noninferiority to laparoscopic Palomo with fewer hydroceles. Radiation exposure during antegrade sclerotherapy in teenagers is measurable yet low, which supports selective use with careful dose control [21,36,80,81].

### 6.4. Impact on Fertility Outcomes

Among operative options, microscopic subinguinal repair has the strongest aggregate signal for improving fertility. A Cochrane review concluded that microscopic subinguinal varicocelectomy probably increases pregnancy rates modestly compared with other surgical approaches. The relative risk was about one point one eight with a confidence interval from one point zero two to one point three six. The same review found that evidence did not clearly favor surgery over embolization for live birth or pregnancy when procedures were compared head to head, reflecting low certainty and heterogeneous trials [3].

In men with recurrent lesions, treating the reflux rather than observing it is associated with higher chances of conception and with consistent gains in semen quality. The systematic review devoted to persistent and recurrent disease reported high anatomical success, clinically meaningful symptom relief, and pregnancy in a substantial fraction of couples within twelve months of redo intervention [54].

### 6.5. Complications and Risk of Second Failure

Safety profiles differ subtly between modalities. Meta-analysis comparing surgical ligation with sclero-embolization across adult and pediatric cohorts found no significant difference in overall complications, yet patterns diverged. Hydrocele was more frequent after surgery, especially when lymphatics were not spared, while epididymo-orchitis occurred somewhat more often after sclero-embolization. Recurrence or persistence rates were broadly similar between groups in aggregate analyses, although some recent pooling suggests a small relative increase in recurrence after embolization when the comparator is high surgical ligation. The absolute differences are modest and are likely outweighed by operator expertise and case selection [82,83].

Microsurgical salvage has a very low risk of hydrocele and arterial injury because lymphatic and arterial structures are preserved under high magnification. In the redo meta-analysis, wound infections and hematomas were infrequent, and the overall probability of a second failure after a well-executed microsurgical redo was small [83].

### 6.6. Special Considerations in Adolescents

In adolescents, management principles mirror those in adults but with greater emphasis on testicular growth trajectories, avoidance of hydrocele, and minimizing radiation exposure [84]. After repair or salvage, testicular catch-up growth may occur over months, and serial volume measurements are more informative than a single postoperative snapshot [85]. When a recurrent varicocele is suspected and testicular catch-up growth is incomplete, a reasonable approach is to observe with structured follow-up for approximately 6–12 months, repeating examination and ultrasonographic volume assessment [86]. Reintervention is commonly considered when asymmetry persists or progresses on serial measurements, particularly when the testicular volume discrepancy remains ≥20% (or >2 mL) and Doppler confirms persistent reflux [66]. This follow-up strategy is strengthened by evidence that a subset of adolescents demonstrates catch-up growth even with observation, supporting individualized decisions in borderline cases [36,81,86].

### 6.7. Practical Synthesis

Retreatment should be individualized and should begin by linking the indication for repeat intervention, such as persistent infertility with abnormal semen parameters, orchialgia attributable to reflux, or impaired testicular growth, to objective confirmation of an active and clinically meaningful varicocele. In men requiring salvage, an artery and lymphatic sparing subinguinal microsurgical redo can improve semen parameters and may achieve pregnancy in a subset of couples [11,73].

A microsurgical redo is generally favored when the index procedure was performed without magnification or when Doppler suggests cord-level collaterals that are amenable to subinguinal control, whereas laparoscopy can be valuable when the suspected failure mechanism is proximal and a higher operative vantage reduces the need for dissection in scarred inguinal planes. After a properly performed subinguinal microsurgical varicocelectomy, true clinical recurrence is uncommon and may reflect proximal duplication or pelvic collaterals rather than missed cord-level branches. In this setting, diagnostic venography with the option for immediate embolization can represent a pragmatic first salvage step, particularly when ultrasonography does not identify a clear cord-level target and repeat surgery would require dense redissection. Selective internal spermatic venography alone may underestimate recurrence when reflux is predominantly extrafunicular, supporting its role as a problem-solving tool rather than a routine test [87,88,89].

Nutcracker physiology should be considered in recurrent cases with a large left-sided varicocele or suggestive symptoms, since renal venous hypertension may increase recurrence risk and may influence counseling and multidisciplinary referral. Following successful salvage, improvement in semen parameters is common and pregnancy has been reported in a substantial fraction of couples within 12 months, although reported rates are heterogeneous and a proportion of couples still require assisted reproduction, which should be incorporated into preoperative counseling [12,73,89,90].

**Figure 2:**
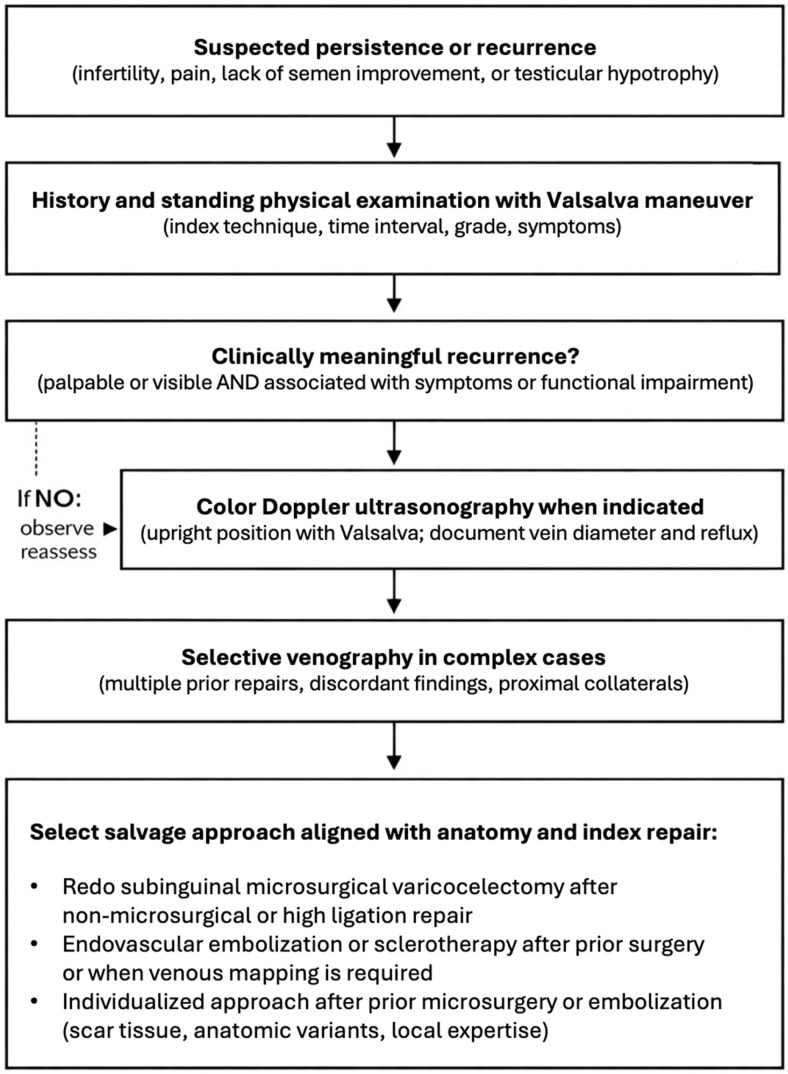
Practical evaluation and salvage framework for suspected persistent or recurrent varicocele. The pathway emphasizes (i) confirming the indication for retreatment, (ii) documenting reflux with clinical examination and Doppler ultrasonography, and (iii) selecting salvage modality based on likely missed anatomy and prior approach, with venography-guided endovascular occlusion prioritized after prior surgery or in complex recurrences and microsurgical redo favored when cord-level collaterals are suspected.

A stepwise evaluation and retreatment framework is proposed in Figure 2.

## 7. Emerging Technologies and Novel Therapies

The therapeutic armamentarium for recurrent varicocele is expanding across robotics, image guidance, endovascular materials and techniques, reconstructive venous surgery, biologic adjuncts, and data-driven decision support. The shared objective is more complete control of reflux pathways with fewer complications, particularly in scarred or anatomically variant fields where first-line methods have failed. Although many innovations remain in early phases, an emerging evidence base from systematic reviews, prospective cohorts, and focused case series helps define where each may add value.

### 7.1. Robotic Assistance

Robotic platforms have been applied to both laparoscopic and subinguinal varicocelectomy, offering stable magnified three-dimensional vision, tremor filtration, and wristed instrumentation that may be advantageous during reoperation. Pediatric and adolescent series establish feasibility and safety, while underscoring a learning curve. In a contemporary experience with robotic approach in children, no intraoperative adverse events were reported, yet early recurrence was not negligible within six months, a signal likely reflecting early adoption rather than inherent limitations of the platform. Comparative pediatric work that directly contrasted robotic and conventional laparoscopy reported similar short-term outcomes, confirming technical parity while leaving questions about long-term superiority unanswered. In adults, small comparative studies suggest robot-assisted subinguinal procedures can reproduce the critical microsurgical steps with outcomes akin to standard microscopy. Cost and access remain limiting, and no study has demonstrated superior fertility outcomes over high-quality microsurgery. As instrumentation miniaturizes and team efficiency improves, robotics may help in redo settings where scar hampers exposure and precision, but at present it should be viewed as an adjunct rather than a replacement for established microsurgery [91,92,93].

### 7.2. Enhanced Intraoperative Imaging

Fluorescent lymphatic mapping with indocyanine green allows real-time identification and preservation of lymphatics and has been associated with very low postoperative hydrocele rates. An initial pediatric series reported identification of all lymphatics with no hydroceles and no recurrences at a maximum of eighteen months, and a later comparative cohort found that indocyanine green lymphography significantly reduced hydrocele formation after laparoscopic Palomo repair. In addition, fluorescence angiography can confirm arterial perfusion after venous ligation during subinguinal microsurgery, supporting arterial preservation when venous branches are secured. These tools, combined with selective intraoperative Doppler ultrasound, can also assist in mapping reflux pathways above and below prior ligation planes during redo procedures, which, in at least one comparative study, translated into more complete vein control and better postoperative motility with no increase in complications. Collectively, image guidance offers practical means to decrease missed channels and lymphatic injury in both adolescents and adults who require salvage surgery [94,95,96].

### 7.3. Interventional Radiology Innovations

Endovascular therapy continues to evolve in ways that are directly relevant to recurrence after surgery. Cyanoacrylate glues such as n-butyl cyanoacrylate can penetrate distal collaterals and create a permanent seal; a systematic review and meta-analysis concluded that glue embolization achieves high technical success and among embolic options tends to show the lowest recurrence at one year, with safety comparable to coils and sclerosing agents. Studies that compared glue with coils reported shorter procedure times and equivalent or lower failure rates when glue was used by experienced operators. Foam sclerotherapy increases endothelium contact and has achieved high technical success in large cohorts, a property that is attractive when a recurrent network contains fine tributaries. Contemporary real-world analyses also show reductions in radiation exposure and cost when sclerosing agents are selected instead of coils. When conventional retrograde access is difficult after prior surgery, operators have reported alternative routes and device strategies to traverse valves and reach high collaterals. Updated standards from the Cardiovascular and Interventional Radiological Society of Europe now codify patient selection, embolic choices, and technical principles aimed at maximizing occlusion and minimizing complications, an important step as centers adopt glue, foam, and modern occlusive devices for complex redo scenarios [21,78].

One distinct advantage of a radiological approach in the redo setting is diagnostic venography. In a postsurgical cohort with persistent varicocele, venography identified venous duplication or alternative channels in a large majority of men and immediate embolization achieved technical success in essentially all cases. This capacity to map and simultaneously treat the culprit pathway is particularly useful when the recurrence stems from high retroperitoneal tributaries that are difficult to expose surgically [77].

### 7.4. Reconstructive Venous Surgery

A microsurgical bypass strategy has been reintroduced that departs from the traditional occlusive paradigm. By anastomosing the internal spermatic vein to the inferior or superficial epigastric vein, surgeons aim to decompress the gonadal venous system by diverting flow into the iliac axis. This approach is conceptually attractive in men with renal venous hypertension due to nutcracker physiology and in selected recurrences where venous hypertension persists despite ligation. A recent comparative series reported that spermatic-epigastric anastomosis was technically feasible in all cases and yielded greater gains in forward motility than standard ligation at one year, albeit with longer operations and hospital stays. Earlier reports, including experience in adolescents, support long-term patency with this anastomosis in carefully selected patients. Evidence remains limited and heterogeneous, and the technique should be considered experimental and confined to centers with microvascular expertise until durable fertility outcomes are established [97,98,99].

### 7.5. Biologic and Pharmacologic Adjuncts

Adjunctive medical therapy seeks to mitigate oxidative stress and inflammation that persist even after reflux is corrected. A systematic review found that antioxidant supplementation after varicocelectomy improves semen parameters at three to six months, though an advantage for pregnancy has not been demonstrated consistently. A recent multicenter randomized trial comparing surgery with an oral antioxidant regimen in men with isolated teratozoospermia and clinical varicocele showed that both strategies improved semen quality and reduced sperm DNA fragmentation, with larger gains and higher natural pregnancy rates after varicocelectomy. In practice, adjuncts such as L-carnitine, vitamins, coenzyme Q-ten, or pentoxifylline may be considered on a case-by-case basis, recognizing that the effect sizes appear modest and that high-quality trials specific to recurrent disease are scarce [100,101,102].

Regenerative approaches are at a preclinical stage yet are increasingly relevant to men with persistent or recurrent disease who have evidence of ongoing oxidative injury. In a controlled rat model of varicocele, injection of conditioned medium from adipose-derived mesenchymal stem cells improved sperm concentration, motility, morphology, and testicular oxidative stress markers to a degree comparable with varicocelectomy, with favorable histology and tight junction protein expression. Animal studies using cellular therapies also show reductions in malondialdehyde and improved spermatogenesis. Translation to human recurrent varicocele has not begun, and key questions remain regarding durability, safety, and integration with mechanical correction of reflux [103,104].

### 7.6. Artificial Intelligence and Data-Driven Care

Machine learning has emerged as a pragmatic tool to refine selection for intervention and to anticipate outcomes. A multi-institutional externally validated model predicted clinically meaningful sperm parameter upgrading after varicocele repair using preoperative clinical, hormonal, ultrasound, and semen data, with performance sufficient for deployment as a counseling aid. Additional models have classified clinical varicocele grade from routine clinical variables with high accuracy and have begun to predict postoperative pregnancy, pointing to broader decision-support roles that could extend to recurrence risk or optimal modality selection when detailed anatomic features and hemodynamics are incorporated. Intraoperative applications are in development, including computer vision to distinguish artery, veins, and lymphatics in real time and navigation support for endovascular access to elusive collaterals, although these uses remain investigational [105,106,107].

### 7.7. Perspective

Across these domains the unifying theme is procedural precision and improved phenotyping. In the near term, fluorescence-guided lymphatic and arterial preservation and modern endovascular materials, supported by standardized technical recommendations, are the most likely to influence routine salvage algorithms because they offer incremental gains within existing surgical and interventional workflows [94,108]. By contrast, robotic platforms remain constrained by cost, access, and limited comparative evidence, and reconstructive venous bypass procedures should remain investigational and confined to specialized centers, potentially in selected patients with persistent renal vein hypertension [109,110]. The most urgent research priorities are methodological: standardizing definitions of persistence and recurrence, integrating hemodynamic classification into preoperative assessment, and reporting patient-centered reproductive outcomes such as time to pregnancy and live birth with sufficiently long follow-up that captures delayed recurrences [54].

## 8. Expert Consensus and Guidelines Perspective

Major urological and reproductive organizations recognize varicocele as a significant contributor to male infertility, yet they provide limited guidance that is specific to recurrence. The scarcity of high-quality evidence devoted to failed repair explains the cautious tone of current recommendations and the reliance on clinician judgment.

The joint guideline of the American Urological Association and the American Society for Reproductive Medicine, most recently updated in 2024, advises repair for infertile men who have a palpable varicocele and abnormal semen parameters when the couple does not face an irreversible female factor. Although the document does not set out a distinct pathway for recurrence, the same criteria are generally applied when a previously treated varicocele remains palpable, and the man is still subfertile. The societies identify microsurgical varicocelectomy as the preferred operative method because it offers low recurrence and complication rates. They also note that repair may induce the appearance of sperm in the ejaculate in selected men with non-obstructive azoospermia, which underscores the potential benefit even in severe presentations. For men with recurrent disease, radiological embolization is presented as a reasonable alternative when prior surgery has failed or when another operation is undesirable [111].

The European Association of Urology takes a similar stance. Treatment is recommended for infertile men with a palpable lesion and impaired semen quality, with explicit endorsement of subinguinal microsurgical ligation as the approach with the lowest likelihood of relapse. For persistent or recurrent cases, the European guidance supports radiological embolization, especially after prior surgery, and suggests that the salvage method should reflect the initial technique. In practical terms, a high ligation failure often prompts a subinguinal microsurgical redo, whereas failure after subinguinal repair may be managed with embolization or an inguinal approach. The European document also advises against routine ultrasonography after repair in asymptomatic men to avoid overtreatment of insignificant residual reflux [2].

The Korean Society for Sexual Medicine and Andrology offers practice-oriented recommendations that mirror this crossover strategy. Management of recurrence is linked to the index procedure, with preference for subinguinal repair after high ligation and for embolization or inguinal ligation after subinguinal repair. The panel cautions that embolization tends to have higher recurrence than microsurgery and therefore reserves it primarily for recurrent disease or specific clinical contexts. The Korean statement emphasizes the importance of surgeon experience and encourages referral to centers with expertise for complex salvage cases [27].

Expert reviews by leading andrologists consistently favor microsurgical techniques for any varicocele because of superior durability and safety. For recurrences, many authors recommend referral to a microsurgeon when the original operation did not use magnification. After repeated failures, the probability of further benefit diminishes, and transition to in vitro fertilization or intracytoplasmic sperm injection may be reasonable, although no formal consensus defines a limit on the number of attempts [112,113].

No organization has produced a dedicated guideline focused exclusively on recurrent varicocele. Recommendations are embedded within broader guidance on varicocele and male infertility, and major evidence syntheses primarily address initial treatment rather than failed repair. Variability in definitions of persistence and recurrence and the use of heterogeneous outcomes such as pregnancy rates, time to conception, and changes in semen parameters further limit the precision of current advice [114].

The Global Andrology Forum published a clinical guide in 2021 that broadly aligns with the positions of the American and European bodies. It advocates a thorough reassessment after an unsuccessful repair, acknowledges that many men still benefit from intervention, and stresses the importance of discussing assisted reproductive options as part of shared decision-making [69].

Taken together, these statements point to a pragmatic framework. Treat clinically significant recurrences using the most effective technique available, match the salvage method to the index approach, and tailor decisions to anatomy, operator expertise, and the couple’s reproductive goals. As research devoted specifically to failed repair accumulates, future guidance is likely to articulate more explicit pathways for recurrent varicocele. Until then, multidisciplinary discussion among urologists, andrologists, interventional radiologists, and reproductive specialists remains essential in complex cases.

## 9. Conclusions

Most persistent or recurrent varicoceles reflect ongoing reflux through missed venous anatomy or unrecognized hemodynamic pathways. Evaluation should be structured: confirm a clinically meaningful recurrence and its indication, document reflux selectively with Doppler ultrasound, and use venography when anatomy is uncertain or prior surgery makes re-exploration high risk. Salvage is effective when aligned with the failure mechanism, with microsurgical subinguinal redo favored for cord-level collaterals after non-microscopic repair and venography-guided embolization preferred for proximal or complex circuits, particularly after prior surgery. Future studies should adopt standardized definitions and emphasize live birth and time-to-pregnancy as primary endpoints.

## Figures and Tables

**Table 1:** Approximate recurrence rates by varicocele repair technique.

Initial Treatment Method	Reported Recurrence Rate	Notes
Microsurgical subinguinal ligation	~0.6–2.5%	Lowest recurrence rates; nearly all variceal veins are visually identified and ligated under high magnification. Large series report recurrence around 1% or lower with this technique [43].
Open inguinal ligation (Ivanissevich)	~5–15%	Higher recurrence if no microscope/magnification is used. Surgeon experience is critical—historically, recurrence ~15% in standard (naked-eye) repairs [43], whereas use of optical magnification (loupes) significantly lowers this rate (to low single digits) [44].
Open retroperitoneal ligation (Palomo)	~9–16%	The high ligation (Palomo) approach can miss collateral veins (e.g., cremasteric), especially when the testicular artery is preserved [45]. Including the artery in the ligation (classic Palomo) reduces recurrence to ~1–5% but is associated with ~30% incidence of hydrocele due to lymphatic disruption [46].
Laparoscopic varicocelectomy	~1–10%	Artery-sparing laparoscopic techniques achieve very low recurrence (often ~1–5%) [46], comparable to microsurgical outcomes. Overall reported recurrence rates range up to ~10% (0–12% in meta-analyses) depending on technique and thorough collateral vein ligation [35]. Proper identification and ligation of all venous collaterals is key to minimizing recurrence.
Percutaneous embolization	~3–13%	Recurrence/persistence varies with venous anatomy, embolic agent, and technical success [47]. Contemporary series commonly report low single-digit to low double-digit recurrence; catheterization failure or recanalization contributes to persistent or recurrent reflux [48].
Antegrade scrotal sclerotherapy	~6–20%	Often used for recurrence; reported success rates are commonly 80–94% [49]. Intraoperative venography and careful technique can reduce persistence, but results vary across series [50].

## Data Availability

No new data were created or analyzed in this study. Data sharing is not applicable to this article.
